# Comments on “The Principle of Least Action for Reversible Thermodynamic Processes and Cycles”, *Entropy* 2018, *20*, 542

**DOI:** 10.3390/e20120980

**Published:** 2018-12-17

**Authors:** Edward Bormashenko

**Affiliations:** Engineering Faculty, Chemical Engineering, Biotechnology and Materials Department, Ariel University, P.O.B. 3, 407000 Ariel, Israel; edward@ariel.ac.il

**Keywords:** heat engines, principle of least action, Carnot cycle, entropy production, transversality conditions

## Abstract

The goal of this comment note is to express my concerns about the recent paper by Tian Zhao et al. (*Entropy* **2018**, *20*, 542). It is foreseen that this comment will stimulate a fruitful discussion of the issues involved. The principle of the least thermodynamic action is applicable for the analysis of the Carnot cycle using the entropy (not heat) generation extrema theorem. The transversality conditions of the variational problem provide the rectangular shape of the *ST* diagram for the Carnot cycle.

The above paper by Tian Zhao et al. [1] argues that the variational principle of least action may be successfully applied for the analysis of heat engines, in the way in which the famous fastest descent “brachistochrone problem” was solved. The authors defined “the optimal process as that which absorbs the maximum heat and outputs the maximum work”, and suggested the determination of which path absorbs the largest amount of heat. This is the main problematic issue of the manuscript. The variational principle in the “brachistochrone problem” enables the derivation of the pathway supplying the minimum to the time span of descent, between a point A and a lower point B, where B is not directly below A, on which a bead slides frictionlessly under the influence of a uniform gravitational field [2,3]. The variational principle says nothing about the maximal time of such a movement [2,3]. Indeed, there exists an infinity of pathways supplying the infinite time of the prescribed motion. Consequently, there is an infinity of thermal (*T*, *S*) pathways supplying the maximum heat to the system. Thus, the principle of the least action was applied by the authors erroneously. Consider also that the time of the Carnot cycle, avoiding irreversible processes, is infinite. Hence, an accurate variational treatment of the Carnot cycle is far from trivial. However, this becomes possible when irreversible thermodynamic considerations are involved [2,4]. 

The variational approach to the Carnot engine based on the geometrical considerations (namely exploiting the transversality conditions of variational problems [2,4]) is also possible. Consider an arbitrary thermal engine following the thermal cycle, depicted in Figure 1 with a blue solid line; the efficiency of the cycle *η* is given by Equation (1): 

The cycle starts at the point (*T*_1_, *S*_1_) and proceeds to the point (*T*_2_, *S*_2_); heat *Q*_1_ is absorbed by the working medium of the engine, and heat *Q*_2_ flows out of the engine.

(1) $$\eta =1-\frac{Q_{2}}{Q_{1}}=1-\frac{\int_{S_{1}}^{S_{2}}T_{II}\left(S\right)dS}{\int_{S_{1}}^{S_{2}}T_{I}\left(S\right)dS}$$

where the integral in the numerator of the ratio (*Q*_1_) is calculated along the thermal pathway denoted in Figure 1 with “*I*”, whereas the integral appearing in the denominator (*Q*_2_), is calculated along the thermal pathway denoted with “*II*”. The maximal efficiency of the engine corresponds to the cycle, for which the ratio $\frac{Q_{2}}{Q_{1}}=\frac{\int_{S_{1}}^{S_{2}}T_{I}\left(S\right)dS}{\int_{S_{1}}^{S_{2}}T_{II}\left(S\right)dS}$ is minimal. Thus, the ratio $\frac{Q_{2}}{Q_{1}}$, should be minimized. This demand is very different from the “maximization of heat”, suggested in [1]. Now, imposing the following physical restrictions:

(2a) $$T_{2}<T<T_{1}$$

(2b) $$S_{2}<S<S_{1}$$

These restrictions are extremely important; indeed, the working media cannot be hotter than the hot thermal reservoir, and it cannot be cooler than the cold one (surrounding medium). It is obvious from the simple geometrical considerations (considering the areas covered by the curves) that the ratio $\frac{Q_{2}}{Q_{1}}$ is minimal under the restrictions, imposed by Equation (2a) and (2b), for the “rectangular” *ST* cycle, shown in Figure 1 with a red dotted line, immediately giving rise to the famous Carnot formula. The right-angles inherent for this “rectangular” cycle (shown with the dashed line in Figure 1) illustrate the transversality conditions of the variational problem [2,4], which should in our case be correctly formulated as follows: when the thermal *ST* pathway “*I*” is given, the pathway corresponding to the minimal possible *Q*_2_ should be necessarily transversal to the curve, describing the pathway “*I*”. 

It is also noteworthy that the efficiency of the Carnot cycle has already been derived successfully with the principle of the least thermodynamic action [5]. Lucia in [5], in order to analyze the Carnot’s efficiency with the variational calculus, exploited Gyarmati’s results, reporting the thermodynamic Lagrangian density [6], immediately yielding the Carnot formula for the optimal heat engine, using the entropy generation extrema theorem, annulling the terms related to irreversibility and dissipation [6]. Consider also that the maximum rate of entropy (not heat) production occurs when all the forces in the system are kept constant. On the other hand, the minimum rate of entropy (again, not heat) production occurs when all the currents that cross the system are kept constant [7,8]. 

It should also be mentioned that the dimension of “thermodynamic action” suggested in [1] is $K^{-1}$, which is quite obscure and is not defined as a mathematic function as introduced in [5]. 

## Figures and Tables

**Figure 1:**
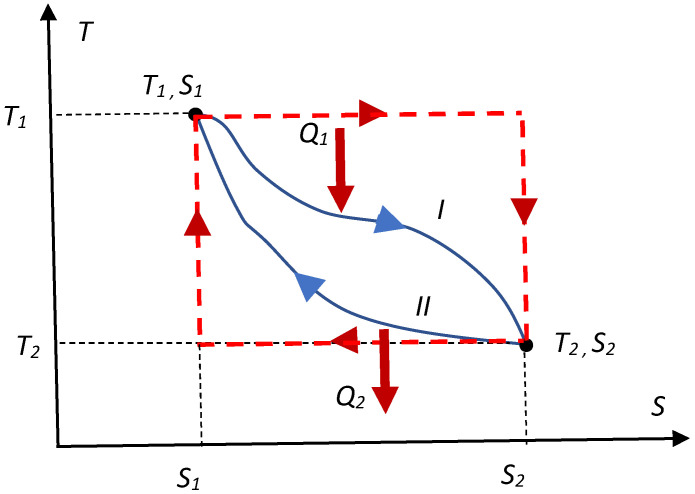
*ST* diagram of the arbitrary thermal cycle, shown with a blue solid line. Red dashed line depicts the Carnot cycle.
